# Undergraduate nurses reflections on Whatsapp use in improving primary health care education

**DOI:** 10.4102/curationis.v38i2.1512

**Published:** 2015-08-13

**Authors:** Juliana J. Willemse

**Affiliations:** 1 School of Nursing, University of the Western Cape, South Africa

## Abstract

**Background:**

The global use of mobile devices with their connectivity capacity, and integrated with the affordances of social media networks, provides a resource-rich platform for innovative student-directed learning experiences.

**Objective:**

The objective of this study was to review the experiences of undergraduate nurses on the improvement of primary health care education at a School of Nursing at a University in the Western Cape, South Africa, through the incorporation of a social media application, WhatsApp.

**Method:**

A qualitative, exploratory, descriptive, and contextual design was used to explore and describe data collected from a purposive sample of 21 undergraduate nursing students. The study population was engaged in a WhatsApp discussion group to enhance their integration of theory and clinical practice of the health assessment competency of the Primary Health Care Module. Participants submitted electronic reflections on their experiences in the WhatsApp discussion group via email on completion of the study. Thematic analysis of the qualitative data collected was done according to Tesch's (1990) steps of descriptive data analysis in order to identify the major themes in the study. The electronic reflections were analysed to explore their rich, reflective data.

**Results:**

Seven themes were identified that included: positive experiences using the WhatsApp group; the usefulness of WhatsApp for integrating theory and clinical practice; the availability of resources for test preparation; opportunity for clarification; anonymity; exclusion of students as a result of the lack of an appropriate device, and the application caused the battery of the device to run flat quickly.

**Conclusion:**

The results of the experiences of students in the WhatsApp discussion group could be used to inform the use of social media applications in teaching and learning, with the purpose of enhancing the integration of the theory and clinical practice.

## Introduction

Emerging technologies, including the use of social media applications such as WhatsApp, are becoming more frequently used in higher education pedagogies; this is also true for the health sciences (Bozalek et al. 2015:2). In this technologically advanced era, it is difficult and sometimes challenging not to be ‘tech-savvy’ as an educator at a Higher Education Institution (HEI).

The global advancement in science and technology makes it difficult to remain indifferent to the presence of technology when individuals are singing the praises of the many technological gadgets that they are using on a daily basis. Madeira et al. (2009:2441), for example, report that emerging technologies afford students the opportunity to visualise and interact with learning content by using multimedia, rich graphics, animation, simulation, and virtual environments. Whilst some studies have been conducted about the use of social media for higher education pedagogies, little has been written with regard to the perspectives of learners about these tools. There is therefore a need to investigate the experiences of students in a WhatsApp discussion group to better ascertain how it might be used to enhance student learning.

This article set out to investigate undergraduate nursing students’ experiences of using WhatsApp to integrate theory and practice of the health assessment in a Primary Health Care Module. A discussion of the investigation follows.

## Studies investigating emerging technologies and mobile learning

Studies about the use of emerging technologies and mobile learning to improve student learning are becoming more prolific in higher education (Bozalek et al. 2015; Pimmer & Pachler 2014; Veletsianos 2010). Technology, such as mobile devices, has permeated our daily lives and provides inexhaustible access to communication and information. Educators and students use mobile technology in diverse contexts for a variety of teaching and learning purposes, for example discussion forums and distribution of content (Conejar & Kim 2014:193–197).An inter-institutional research project funded by the National Research Foundation in South Africa was initiated in 2011 at 22 public South African HEIs. The purpose of this was to explore the use of emerging technologies to transform teaching and learning interactions and paradigms (Gachago et al. 2013:95). As part of the study, the perceptions of the meaning that the term ‘emerging technologies’ had for both participants and the team of 16 researchers from South African HEIs were investigated. Seven characteristics of the concepts in relation to emerging technologies were identified as part of the study by the research team and research participants. One of these characteristics was particularly appropriate for this study about students’ experiences in relation to WhatsApp, namely Characteristic 7: ‘Emerging technologies provide personalized learning opportunities’ (Gachago et al. 2013:98).

Another study at the Faculty of Communication and Information Science at the National University of Science and Technology in Bulawayo, Zimbabwe investigated the extent of mobile phone access and usage to support information sharing amongst students (Dewah & Mutula 2013:150). The study concluded that most of the students acquired a mobile phone that had the ability to support their studies and their continued communication with their family members. It emerged that students’ phones had functionalities and services, including short messaging services (SMSs), Internet, memory card ports, multimedia messaging, as well as call waiting and forwarding services. Furthermore, the study reported that students used their mobile phones to share academic information with their peers and to make appointments with their educators to discuss academic work. However, the authors suggested that regulations have to be implemented for the use of mobile phones. Dewah and Mutula (2013:162) acknowledged that regulations have to be implemented for the use of mobile phones during lectures, tests, and examinations.

The potential for using WhatsApp as an instant messaging tool is particularly pertinent in resource-poor contexts, because it is currently one of the most commonly used applications on mobile phones and computers; it is a free service for one year with a minimal fee payable thereafter (Yeboah & Ewur 2014:157). Owing to affordability and accessibility, instant messaging has great potential for use in higher education for both formal and informal learning, particularly in resource-poor contexts such as the HEI where this study was undertaken (Bere 2012; Church & De Oliviera 2013; Ng’ambi & Bozalek n.d.). WhatsApp and other instant messaging applications have been found to be beneficial for learning because they increase student participation in both face-to-face and distant contexts between students, other students, and educators (Johnson et al. 2015; Makoe 2012; Nicholson 2002; Rambe & Bere 2013).

This study was theoretically positioned in John Dewey's (1944) theoretical contributions to learning and was guided by two approaches to learning within those broad theoretical parameters, namely authentic and flexible learning. Those approaches were used to provide a foundation for the pedagogical activities whilst using a social media application on a mobile device (Herrington et al. 2009:4).

The increased impact of constructivism as a philosophical approach to learning has prompted many educators at universities to implement more ‘authentic’ teaching and learning environments (Herrington & Herrington 2006:2–3). In this study, an ‘authentic’ teaching and learning environment was created by using a social media application, WhatsApp, to create learning opportunities for students that enabled them to benefit and tap into once they had left the learning institution to apply the theory at clinical practice facilities (Herrington, Reeves & Oliver 2010:14). The students focused on engaging in innovative and realistic tasks that afforded opportunities of complex collaborative actions for authentic learning to occur (Herrington et al. 2010:18).

Flexible learning is a set of educational philosophies and systems that seek to provide students with increased choice, convenience, and personalisation to suit each individual student. In particular, flexible learning provided students with choices about where, when, and how their learning occurred (Shurville, O’ Grady & Mayall 2008:74–84).With the advent of PHC, client care has shifted from a hospital to a community-based environment that requires educators to focus on new approaches and tools to support their teaching and learning in the autonomous and diverse practice environment, where resources are not readily accessible (Kenny et al. 2009:76). Because students in this study ‘worked’ at clinical facilities for two days per week, educators had to find ‘alternatives’ to their teaching strategies, with the aim of affording students the opportunity to learn where, when, and how they could manage their learning. Students were able to communicate questions in relation to their experiences at clinical facilities to get clarification from their educators when they were not present at the clinical facility. It gave them access to an ‘e-educator’ wherever, whenever and however clarification and guidance was needed.

John Dewey (1944), a realist and experiential learning theorist, emphasises the value for students who gain knowledge through personal experiences (Bruce, Klopper & Mellish 2011:169). This study afforded students an experience of using a social media application on their mobile devices to integrate their theory and clinical practice whilst doing the health assessment of the Primary Health Care Module. The experiential learning process allowed knowledge generation through the conversion of experiences in the WhatsApp discussion group (Lai et al. 2007:326).

This study explored the effectiveness of improving knowledge creation during experiential learning activities with the use of technology as a method of instruction in teaching (Lai et al. 2007:326; Pitler et al. 2007:2). The use of technology in teaching provided a learning platform that led to a dynamic and exciting learning environment for students who participated in this study (Pitler et al. 2007:2). The use of a social media application on mobile devices provided educators with scope to arrange a comprehensive variation of opportunities for learning to accommodate the different learning needs and capabilities of individual students (Pitler et al. 2007:3).

## Reflection and reflective learning

Reflection and reflective learning are an integral part of experiential learning (Bruce et al. 2011:198). Students experienced experiential learning in the WhatsApp discussion group because it was a method of communication which had not been used before at a school of nursing at a University, where the study was conducted. This was a pure trial and error experience for students who volunteered to be part of the study. The electronic reflections gave students the opportunity to reflect on what they had experienced whilst participating in the WhatsApp discussion group, which was aimed at enhancing the integration of theory and clinical practice for purposes of the health assessment in the Primary Health Care Module.

## Research aim

The study aimed at enhancing and promoting learning through the incorporation of a social media application, WhatsApp, as a mode of communication for enhancing the integration of theory and clinical practice. The study formed part of the health assessment of the Primary Health Care Module. The purpose was to assist participants in ‘real’ time when they were experiencing any challenges with the application, and required guidance from educators and peers whilst at clinical facilities.

## Research method and design

A qualitative, explanatory, descriptive, and contextual design was applied to explore the integration of theory and clinical practice of the health assessment competency in the Primary Health Care Module of the third year undergraduate nursing programme by using WhatsApp, a social media application, on students’ personal mobile devices.

## Research setting

The research study was conducted in the context of an undergraduate nursing programme at an HEI in the Western Cape Province.

## Research population and sample

The accessible population of this study included students registered for the Primary Health Care Module, to whom the researcher had reasonable access and who met the inclusion criteria (Brink, Van Der Walt & Van Rensburg 2013:131). To be included in the study, students had to be:

- in possession of a personal mobile device with access to WhatsApp, a social media application
- prepared to use their personal mobile device as a tool to take part in the study, and
- registered for the Primary Health Care Module.

A total of 40 students registered for the Primary Health Care Module in semester one in 2014 were invited to an information session to explain the purpose of the study. Twenty-nine students, who met the inclusion criteria and gave voluntary consent to be part of the study, made up the total population of this study.

## Ethical considerations

Ethical approval (12/10/16) for this study was obtained from the Senate Higher Degrees Committee of the Faculty of Community Health and Sciences, the Registrar, and the Director of the School of Nursing at the University of the Western Cape prior to the implementation of the study. An information session was arranged with students before written informed consent was obtained. Participants were informed that participation was voluntary. Participants consented to have their cell phone numbers made available to the researcher, who undertook not share it with any other person, except with the research supervisor.

### Data collection

Students who had volunteered and met the inclusion criteria participated in the WhatsApp discussion group for seven weeks during term one of 2014 in an attempt to enhance the integration of the theory and clinical practice of the health assessment in the Primary Health Care Module. At the end of the seven weeks in the WhatsApp discussion group, students were requested to reflect on their experiences on completion of the implementation of the study. Twenty-nine students participated in the study, but only 21 students submitted electronic reflections on their experiences in the WhatsApp discussion group via email. Eight students had to withdraw from the study after the allocation of participant numbers as they encountered challenges with their mobile devices. A total of seven reflections were also received from students who had to withdraw from the study and students who never participated in the study in the first place as they did not meet the inclusion criteria. The reflections were based on the limitations experienced as a result of not being part of this group.

### Data analysis

A systematic data analysis was done, guided by Tech's data analysis method (1990) as cited by Creswell (2003:192). The reflections were thematically analysed to develop themes in an inductive way as directed by the content of the data (Braun & Clarke 2006:77). Seven main themes were identified from the reflections of the students based on their experiences in the WhatsApp discussion group.

## Trustworthiness

Four criteria: credibility, dependability, conformability and transferability are suggested by Lincoln and Guba (1985), as cited in Polit and Beck (2012:583), to develop trustworthiness in qualitative inquiry (Polit & Beck 2012:584) and as discussed in Table 1.

**TABLE 1:** Strategies to ensure trustworthiness.

Strategy	Application
Credibility	Ensured through engagement with students within the WhatsApp discussion group and with data during the data analysis process. The researcher and two educators participated in the discussion group with the students. Data analysis was done according to Tesch’ s (1990) steps of descriptive data analysis in order to identify the major themes in the study.
Dependability	A rich description of the research methodology and data analysis. Direct quotations from participants. Description of the ethical principles adhered to.
Conformability	A rich description of the research methodology and data analysis. An independent coder reviewed the accuracy of themes identified from data collected.
Transferability	Inclusion of the actual discussions within the WhatsApp group to provide comprehensive evidence to guide use by other researchers.

## Results and discussion

The main themes identified in the data analysis are presented in Table 2.

**TABLE 2:** Main themes identified.

Theme	Data analysis
Theme 1	Positive experiences using the WhatsApp group
Theme 2	Usefulness of WhatsApp for integrating theory and clinical practice
Theme 3	Availability of resources for test preparation
Theme 4	Opportunity for clarification
Theme 5	Anonymity: Feeling comfortable about being anonymous
Theme 6	Exclusion due to lack of an appropriate device or application
Theme 7	Application caused the battery of the device to run flat quickly

### Positive experiences using the WhatsApp group

Students reflected a positive learning experience in the WhatsApp discussion group and with access to their educators and peers when they needed guidance or information. Positive learning experiences included access to a variety of clinical cases and information, the creation of a learning platform and the availability of educators and peers to answer questions.

‘I had a great experience with the WhatsApp group. It provided me with a variety of clinical cases and access to information that I did not know. It's also something I can use to reflect back on when confronted with similar cases.’ (Participant 7)‘This WhatsApp group was a great learning platform for me, if I was unsure about something, I could always ask our supervisors (clinical facilitators) or lecturer and other students could learn from my question or experience.’ (Participant 17)

WhatsApp became very popular, as the use of the application is free for the first year, after which a small fee is payable to continue with the service, making it affordable and accessible (Yeboah & Ewur 2014:157). In an investigation into the pedagogical suitability of using cell phones to enhance learning, Makoe (2010:99) found that the use of social media applications, such as Mxit and WhatsApp, allowed lecturers to communicate with students and send information at an affordable cost.

### Usefulness of WhatsApp to integrate theory and clinical practice

Students experienced that their WhatsApp discussion group helped them to ‘*grasp*’ how to apply theory during clinical practice, and it served as a platform to support the integration of theory and clinical practice.

‘I honestly feel as if is the first time that I truly grasp the concept of applying theory to practical.’ (Participant 3)‘The WhatsApp group served as a valuable platform which allowed me to integrate my theoretical knowledge … into my practical experience in the clinical setting. I honestly feel as if is the first time that I truly grasp the concept of applying theory to practice. I learnt to share my knowledge with others and in return gain from their experiences.’ (Participant 10)

In 2011 and 2012 smartphones became the most globally used connected computer devices, when smartphone ownership started to outnumber basic cell phone ownership (Cochrane 2014:65). Emerging technology affords students the opportunity to visualise and interact with learning content by using multimedia, rich graphics, animation, simulation, and virtual environments (Madeira et al. 2009:2441). The global possession and connectivity of mobile devices, integrated with the affordances of social media networks, provide a resource-rich platform for innovative, student-directed learning experiences (Cochrane et al. 2014:1). The affordances of mobile technology create an opportunity to visualise and interact with learning as demonstrated in the extract from a WhatsApp conversation presented in Box 1.

**BOX 1:** Extracts from a WhatsApp conversation.

12:19, 24 Feb – **Educator 1**: Dear students. Tonsillitis is a common condition managed at a primary level of care. Have you managed a client with tonsillitis as yet? Oh yes … What types of tonsillitis have you ‘seen‘ and what clinical picture did your client present with? 14:55, 24 Feb – **Participant**: The patient came in complaining of pain on swallowing for six days. On inspection, the tonsils were very swollen. On palpation, the tonsillar lymph nodes were enlarged. No visible white patches though. Is this bacterial or viral tonsillitis? 14:57, 24 Feb - **Participant:** It's viral if the [there] are no white patches. 14:58, 24 Feb - **Participant:** Bacterial tonsilitis [tonsillitis] usually presents with white patches or pus on the tonsils. 15:02, 24 Feb - **Participant:** Its viral tonsillitis [tonsillitis]. Bacterial tonsilitis [tonsillitis] associated with pus and white patches. 15:03, 24 Feb - **Participant:** Has the patient have a fever?
15:18, 24 Feb - **Educator 2**: Bacterial tonsilitis [tonsillitis] is mainly caused by the bacterium called streptoccocus [streptococcus]. There is high fever, difficulty in swallowing, and enlarged tonsils, sometimes with yellowish patches. The lymph glands at the angle between the lower jaw bone and the neck get enlarged and become painful. In virus tonsilitis [tonsillitis], yellowish patches do not occur, the fever is not that high, lymph gland enlargement in the neck is not so prominent and not so painful. 16:01, 24 Feb - **Participant:** Why must you give saltwater gargles and not antiseptic gargle? 16:04, 24 Feb - **Participant:** You can give antiseptic but salt water is more accessible. 16:08, 24 Feb - **Participant:** Saltwater could also be less harsh on the already sensitive/raw throat. 16:12, 24 Feb - **Educator 2**: Antiseptic gargles should not be given as it destroys the normal flora of the mouth and has NO effect on viruses.

The above extracts from the WhatsApp conversations support the integrative process and provide an indication of how learning took place during discussions in the group.

### Availability of resources for test preparation

Participants reflected that the WhatsApp discussions created an online discussion trail that allowed them to go back to information and use it in preparation for assessments.

‘It is easy to study for [*the*] test because we have discussed in the group that is why it is very important.’ (Participant 4)

‘I find it very helpful, I was confident enough when I was doing my evaluation because I know what to look for because we already covered that on [*sic*] WhatsApp group …’ (Participant 23)

Currently, WhatsApp can be regarded as the cross platform between the instant messaging application and mobile instant messaging (MIM) on smartphones (Church & Oliveira 2013:352). The study participants of Church and Oliveira (2013:354) indicated that they sent more messages using WhatsApp compared to SMSs because they were not limited regarding the number of characters and content on WhatsApp. WhatsApp allows the sending and receiving of information, images, video, audio, and text messages in ‘real time’ to individuals and groups (Church & Oliveira 2013:352; Lai et al. 2007:326).

### Opportunity for clarification

Participants appreciated the presence of their educators in the group because they were able to provide them with guidance in decision making, when required:

‘It was nice the guidance of (to be guided by) our supervisors and lecturers.’ (Participant 1)

‘Personally, I used it to confirm what was said in class or by my supervisor.’ (Participant 25)

Effective learning is dependent on the abilities of the lecturers’ and clinical facilitators’ abilities to encourage and support collaborative learning (Makoe 2012:94).

### Anonymity: Feeling comfortable about being anonymous

Participants reflected on the ability to post a question or a comment without feeling intimidated, because no one knew their identity in the group. The only detail that reflected in the discussion group was the participants’ mobile device numbers; their true identity was only known to the researcher.

‘I liked the fact that it was anonymous, so it gave me the freedom to ask anything without the fear of being criticised without it feeling as if I’m asking a “stupid” question.’ (Participant 11)

**FIGURE 1:**
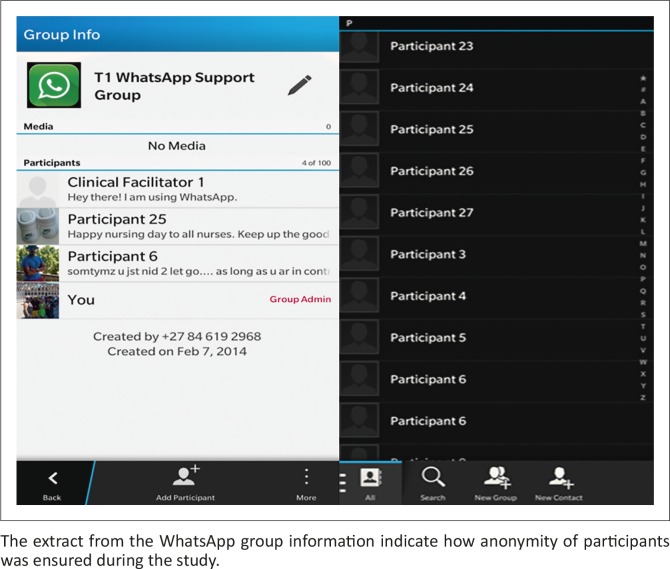
Anonymity within the WhatsApp group. The extract from the WhatsApp group information indicate how anonymity of participants was ensured during the study.

Anonymity of participants strengthened the notion of asking a question without having to feel intimidated as no one could identify an individual within the group discussion as illustrated in Figure 1.

The above extract from the WhatsApp group information indicates that the anonymity of participants was ensured during the study.

Warner et al. (2011:1065) assessed the influence of anonymity on screening processes in relation to the willingness of soldiers to report mental health problems during a post-deployment health assessment. On the grounds of their comparative analysis of anonymous and non-anonymous surveys, this study concluded that anonymity encouraged honest reporting.

### Exclusion due to lack of an appropriate device or application

Students were invited to voluntarily participate in the WhatsApp discussion group, but unfortunately there were students who did not meet the inclusion criteria of either possessing a mobile device or being able to access the social media application, WhatsApp. The students had, however, been accommodated with a weekly overview of the WhatsApp discussions of the past week before the next lecture session commenced. One participant in the study voiced a concern that the contribution to the discussions by students who were not part of the group was ‘*lost’.*

‘Not all students were able to access the group. So, we lost their participation.’ (Participant 24)

‘Not having WhatsApp made it difficult to communicate with the WhatsApp group.’ (Non-participant)

‘Unfortunately, I was not part of the WhatsApp group because my phone does not have access to [*the*] Internet. I struggled a lot but through hard work and help from my clinically supervisor (facilitator) I managed.’ (Non-participant)

‘I did not have [*the*] WhatsApp application because of various reasons. I did not want to be left out so I used my friends’ who are on WhatsApp to help me to keep me updated about every discussion they had on WhatsApp.’ (Non-participant)

A review of the literature by Cochrane et al. (2014:2) recognises the existence of a few well-developed theoretical frameworks to support creative pedagogies where participants have to ‘bring your own device’ (BYOD) in order to be participative. Supporting creative pedagogies using BYOD through the inclusion of collaborative practice with the established teacher communities of practice enhances learning about the affordances of mobile devices in relation to new models of student learning. The affordances of mobile devices, coupled with the collaborative affordances of social media, provide a rich platform for creative student-directed learning experiences.

### Battery of the mobile phone ran flat easily

Participants raised the concern about the battery running flat whilst they were participating or following the discussion of the WhatsApp group:

‘But the problem came when my cell phone battery didn’t last very long using the group.’ (Participant 9)

Amongst the challenges experienced with mobile learning, the limited battery life of mobile devices seems to cause the most distress because it limits the students’ access to educational material whilst on the move (Moldovan, Weibelzahl & Muntean 2014:234).

## Conclusion

The use of WhatsApp, a social media application, provides students with a support structure to enhance the integration of their theory and clinical practice of the health assessment competency in the Primary Health Care Module. The reflections of students highlight their positive experiences of the enhancement of their learning as a result of the intervention. The intervention provides a virtual space for collaborative practice, sharing of course related information, and maintains academic support to enhance the teaching and learning process.

The findings of this study are a contribution towards the usage of social media applications in teaching and learning, with the purpose of enhancing the integration of theory and clinical practice. It could also guide the implementation of an original intervention which incorporates mobile devices and M-learning into a programme to enhance learning within health education.
